# Anemia, Blood Transfusion, and Filter Life Span in Critically Ill Patients Requiring Continuous Renal Replacement Therapy for Acute Kidney Injury: A Case-Control Study

**DOI:** 10.1155/2019/3737083

**Published:** 2019-01-28

**Authors:** Hasan M. Al-Dorzi, Nora Ali Alhumaid, Nouf Hamad Alwelyee, Nouf Mubark Albakheet, Ramah Ibrahim Nazer, Sadal Khalid Aldakhil, Shahad Abdulaziz AlSaif, Nazish Masud

**Affiliations:** ^1^Intensive Care Department, Ministry of National Guard-Health Affairs, Riyadh, Saudi Arabia; ^2^King Abdullah International Medical Research Center, Riyadh, Saudi Arabia; ^3^College of Medicine, King Saud bin Abdulaziz University for Health Sciences, Riyadh, Saudi Arabia; ^4^Department of Medical Education, King Saud bin Abdulaziz University for Health Sciences, Riyadh, Saudi Arabia

## Abstract

**Background:**

Filter clotting is frequent during continuous renal replacement therapy (CRRT), which increases anemia risk. We studied anemia and blood transfusion in critically ill patients requiring CRRT for acute kidney injury and assessed the relationship between CRRT filter life span and PRBC transfusion.

**Methods:**

A case-control study was conducted at a tertiary-care intensive care unit (ICU) where CRRT cases were matched with controls for age, gender, admission category, and severity of illness. Daily hemoglobin levels, blood transfusions, and life span of CRRT filter were noted. CCRT patients were categorized according to the median of the filter life span (20 hours).

**Results:**

Ninety-five cases and 102 controls were enrolled. The hemoglobin level on admission was similar in the two groups, yet cases had significantly lower hemoglobin levels than controls (72.8 ± 15.3 versus 82.5 ± 20.7 g/L, *p* < 0.001) during ICU stay. Anemia <70 g/L occurred in 50% of cases and 19% of controls (*p* < 0.001). Most (56.3%) cases were transfused compared with 29.9% for controls (*p* < 0.001) with higher number of transfused packed red blood cell (PRBC) units in cases (2.6 ± 4.0 versus 1.5 ± 3.2 units per patient, *p*=0.03). Patients with shorter versus longer filter life had similar hemoglobin level in the first 7 days of CRRT with no difference in PRBC transfusion requirement. Prefilter heparin use and hemodialysis access location were not associated with longer filter life span. The mortality was similar in patients with shorter versus longer filter life.

**Conclusions:**

CRRT in ICU was associated with larger drop in hemoglobin and more PRBC transfusion. Shorter (<20 hours) versus longer CCRT filter life was not associated with increased PRBC transfusion.

## 1. Introduction

Acute kidney injury (AKI) is common in critically ill patients and may develop in about two thirds of the patients admitted to the intensive care unit (ICU) [1]. Continuous renal replacement therapy (CRRT) is required in 4-5% of ICU patients [1, 2] and is considered the preferred AKI treatment in the ICU as it preserves hemodynamic equilibrium by gradually removing fluid and urea [3]. However, CRRT is associated with premature clotting when blood passes through the circuit membranes [4], as a result of a coagulation cascade initiated by a complex interplay between the patient's blood and the circuit [5]. This would lead to CRRT interruption and blood loss, which can reach 300 ml if the blood could not be returned. Blood loss can cause anemia and may increase the requirement for blood transfusion. Clinical studies have shown an association between CRRT and anemia and blood transfusion. One study reported that CRRT was associated with an increased requirement for blood transfusion with a temporal relationship between the occurrence of hemofilter blood clots, which could be reflected by the hemofilter lifespan, and blood transfusion [6]. Another study concluded that premature clotting of the CRRT circuit increased blood loss, workload, and costs [7]. A third study which was conducted in patients with combined liver and renal failure found that CRRT circuit life span was generally short and did not improve even with the use of anticoagulation [8].

Anemia and blood transfusions are associated with increased morbidity and mortality in ICU patients in general [5, 9], which makes the prevention of CRRT circuit clotting an important goal in clinical practice. The use of regional anticoagulants, such as citrate and unfractionated heparin (UFH), is recommended [10]. However, their use is variable, and their impact on clinical outcomes remains unclear. In an attempt to improve the practices of CRRT, we performed this study with the main objective of studying the occurrence of anemia and blood transfusion in ICU patients requiring CRRT in relationship to the circuit life span and outcomes.

## 2. Materials and Methods

### 2.1. Study Design and Setting

This was a case-control study that was conducted in the Intensive Care Department of King Abdulaziz Medical City (KAMC), Riyadh. The hospital had five adult noncardiac ICUs (60 beds) with annual admission of approximately 2000 medical, surgical, and trauma patients. A significant proportion (>25%) of these patients developed AKI [11] and required CRRT (estimated to be approximately 10% of admitted patients). This study was approved by Institutional Review Board of King Abdullah International Medical Research Center.

### 2.2. Selection of Patients

Consecutive sampling technique was used to select cases and controls from the cohort of patients admitted to the ICUs between January 1, 2010, and December 31, 2016. Before obtaining the cases and controls, we excluded patients staying less than 7 days in the ICU, who had hemoglobin level of <90 g/L on ICU admission, or who were on hemodialysis before ICU admission as these variables may affect CRRT use and blood transfusion practices during ICU stay. The cases were adult patients 18 years and above who had CRRT for AKI. During the study period, the Prismaflex® system was used for CRRT according to the manufacturer's guideline by trained critical care nurses at a 1 : 1 nurse-to-patient ratio. The Prismaflex® hemofilter set (M100) was changed after a maximum of 72 hours of use, or as needed. The filter was routinely primed by 5000 IU of UFH. Prefilter heparin was then provided, and its dose titrated as per the discretion of the treating team. During the study period, an algorithm for dosing heparin was not used and regional citrate anticoagulation was not available. Controls were ICU patients who did not receive CRRT matched for age (±2 years), gender, Acute Physiology and Chronic Health Evaluation (APACHE) II score (±2), and admission category (medical, surgical, and trauma).

### 2.3. Data Collection

The main variables extracted for cases and controls included demographics, presence of chronic health illnesses as per APACHE definitions, preexisting hypertension and diabetes, APACHE II score [12], presence of sepsis and shock within 24 hours of ICU admission, requirement for mechanical ventilation, hemoglobin level on admission to the ICU and the nadir hemoglobin level during ICU stay, admission creatinine and lactate, CRRT use and duration, location of hemodialysis access, number of filters used, life time of each filter, use of intravenous heparin infusion to prolong filter life, and the number of units of transfused packed red blood cells (PRBC). For cases, we also noted during the first seven days of CRRT treatment, the daily hemoglobin level, the highest international normalized ratio (INR) of the prothrombin time and activated partial thromboplastin time (aPTT), the lowest platelet count, and the number of units of transfused platelets and fresh frozen plasma.

The primary outcome variable was the occurrence of anemia and requirement for blood transfusion. According to the World Health Organization, anemia is defined as hemoglobin <130 g/L in men who are >15 years old and <120 g/L in nonpregnant women who are >15 years old [13]. As hemoglobin <100 g/L is a clinically more relevant cutoff than <120 g/L and is frequently used to determine the need for blood transfusion [9, 14, 15], we defined the development of anemia when nadir hemoglobin was <100 g/L. We also assessed the occurrence of anemia with hemoglobin <70 g/L, the threshold for restrictive blood transfusion in the ICU setting [15]. We also studied ICU and hospital mortality, bleeding during ICU stay, duration of mechanical ventilation, and ICU and hospital length of stay.

### 2.4. Statistical Analysis

Statistical analysis was done using Statistical Software for Social Sciences (SPSS) version 21. Categorical variables were presented as frequencies and percentages and were compared using the chi-squared test. Continuous variables were reported as means with standard deviation and were compared using Student's *t*-test. For certain variables, we also presented the median and the interquartile range. Multivariable logistic regression analysis was performed to determine if CRRT for AKI was associated with drop in hemoglobin to <70 g/L during ICU stay. Variables entered in the model were those with *p* < 0.20 between cases and controls (age, body mass index, chronic cardiac, renal and hepatic illnesses, presence of sepsis, presence of shock, and admission serum creatinine and lactate). To assess the factors associated with filter clotting, cases were divided by filter life span median (calculated to be 20 hours) into shorter versus longer filter life span and their characteristics were compared. Multivariable logistic regression analysis was also performed to assess the predictors of shorter versus longer filter life. Variables entered in the model were those with *p* < 0.20 between patients with shorter versus longer filter life span (body mass index, admission category, sepsis on admission, and serum creatinine on CRRT start) in addition to prefilter heparin use and location of dialysis access. The results of regression analyses were presented as odds ratio (OR) with 95% confidence interval (CI). We also studied the correlation between the starting heparin dose and the first filter life span using linear correlation analysis. For all the statistical tests, *p* value of <0.05 was considered significant.

## 3. Results

### 3.1. Characteristics of Patients

A total of 197 patients were enrolled; 95 patients who had CRRT in the ICU (cases) and 102 controls. The demographic and baseline clinical data of the study participants are summarized in Table 1. There were no significant differences between the two groups in the variables used for matching cases and controls (age, gender, and APACHE II score). Sepsis and shock on admission, which predispose for AKI, were significantly higher in the CRRT patients (*p*=0.002  and < 0.001, respectively). As expected, admission creatinine was significantly higher in the CRRT group.

### 3.2. Anemia and Blood Transfusion

The mean hemoglobin on ICU admission was similar in cases and controls (111.8 ± 18.7 and 113.6 ± 26.6 g/L, respectively, *p*=0.60). However, the lowest recorded hemoglobin during stay was significantly lower in cases (72.8 ± 15.3 g/L) than controls (82.5 ± 20.7 g/L, *p* < 0.001). Anemia with hemoglobin <100 g/L was very common in cases (92.7%) and controls (82.2%; *p*=0.03). Anemia with hemoglobin dropping <70 g/L occurred in 49 (50%) cases compared with 19 (19%) controls (*p* < 0.01). More patients in the CRRT group (*N* = 54, 56.3%) required PRBC transfusion compared with controls (*N* = 30, 29.9%) (*p* < 0.001). Also, the average number of transfused PRBC was significantly higher in the CRRT group (1.5 ± 3.2 versus 2.6 ± 4.0 units per patient; *p*=0.03). PRBC transfusion in response to hemoglobin drop may have contributed to no significant difference in hemoglobin difference between cases and controls.

On multivariable logistic regression analysis, CRRT for AKI was the only variable associated with drop in hemoglobin <70 g/L (OR, 7.09; 95% CI, 3.26–15.41). The *p* value for the Hosmer and Lemeshow test was 0.71. The area under the curve for the Receiver Operating Characteristic C statistic was 0.758 (95% CI, 0.689–0.826). Both tests indicated that the logistic model was a good fit.

### 3.3. Circuit Filter Life Span

A total of 857 filters were used in cases during the study period with an average of 8.9 ± 10.9 filters per patient (median = 6, interquartile range = 3 and 11). The average filter life span per patient was 23.0 ± 13.0 hours (median = 20 hours, interquartile range = 14 and 29 hours). Patients with shorter (<20 hours) versus longer (≥20 hours) filter life had similar characteristics including the highest level of aPTT and INR and the lowest level of platelets (Table 2). Prefilter heparin was provided in 17 (38.6%) patients with filter life <20 hours and 15 (29.4%) patients with filter life ≥20 hours (*p*=0.34).

**Figure 1:**
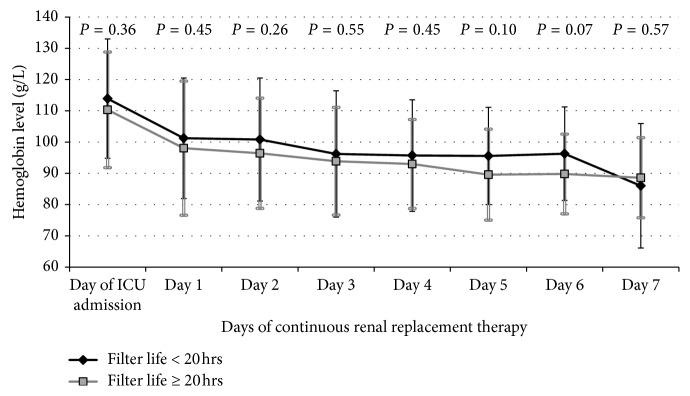
Hemoglobin levels on admission to the intensive care unit and on the first seven days of continuous renal replacement therapy. The error bars represent standard deviation.

Figure 1 describes the nadir hemoglobin on each of the seven studied days on CRRT. There was no significant difference in the hemoglobin level on any of the 7 days between the shorter versus longer filter life groups. The lowest hemoglobin during ICU stay was 76.0 ± 17.2 g/L for patients with shorter filter life compared with 70.5 ± 13.1 g/L for those with longer life span (*p*=0.09). PRBC transfusion was provided in 24 (54.5%) patients with shorter filter span and 29 (56.9%) patients with longer life span (*p*=0.82). The number of transfused packed RBC was similar in the shorter and longer life span groups (2.5 ± 4.3 and 2.7 ± 3.8 units, respectively; *p*=0.81).

On multivariable logistic regression analysis, none of the variables was independently associated with filter life span. Neither regional heparin (OR, 1.10; 95% CI, 0.42–2.87) nor the location of the hemodialysis access (femoral versus internal jugular vein: OR, 1.32; 95% CI, 0.52–3.35; *p*=0.98) was associated with a longer filter life. The *p* value for the Hosmer and Lemeshow test was 0.30. The area under the curve for the Receiver Operating Characteristic C statistic was 0.690 (95% CI, 0.579–0.800). Both tests indicated that the logistic model was a good fit. We also did not find any correlation between the starting heparin dose and the first filter life span (Pearson correlation coefficient = 0.03; *p*=0.91).

### 3.4. Outcomes of Patients

The overall ICU mortality of enrolled patients was 37.1% (55.2% (*N* = 53) for cases and 19.8% (*N* = 20) for controls; *p* < 0.001. The hospital mortality rate was 49.2% (61.5% for cases (*N* = 59) and 37.6% (*N* = 38) for controls; *p*=0.001. Bleeding complication during ICU stay occurred in 8.5% of studied patients with no significant difference between cases (*N* = 10, 10.5%) and controls (*N* = 6, 6.5%; *p*=0.32).

Table 3 presents the outcomes of patients with filter life span <20 hours versus ≥20 hours. The ICU and hospital mortality and bleeding rates were similar. Moreover, both groups had similar mechanical ventilation duration and ICU and hospital length of stay.

## 4. Discussion

In this study, the main findings were as follows: in critically ill patients with AKI requiring CRRT, hemoglobin level dropped to <70 g/L and blood transfusion was provided more often compared with other ICU patients; the median life span of CRRT filters was 20 hours; there was no association of filter life span with anemia or blood transfusion; and neither the use of low-dose IV heparin nor the location of the hemodialysis access was associated with the life span of filter.

Several studies evaluated the epidemiology of anemia in the ICU setting and found that anemia was common in critically ill patients in general and was associated with worse outcomes [9, 16]. Our study assessed anemia in a subgroup of ICU patients and found that half of patients with AKI requiring CRRT (50%) had anemia (hemoglobin <70 g/L) during their course of treatment. CRRT was an independent predictor of drop in hemoglobin to <70 g/L on multivariable logistic regression analysis. Other studies have shown different results. One study observed that baseline anemia (<120 g/L in women and <135 g/L in men) before starting CRRT for AKI was present in 83% with hemoglobin level dropping by 5% in the subsequent 7 days [17]. Another study found that new onset anemia (hemoglobin <100 g/L) during CRRT occurred in 31% [18]. The drop in hemoglobin during CRRT can be attributed to multiple factors including extracorporeal blood loss. Requirement for blood transfusion during CRRT treatment has been variable between different studies. In our study, 56.3% of patients receiving CRRT required blood transfusion. One study reported that 33.7% of such patients required RBCs transfusion during treatment [18]. Another study observed a higher percentage (65%) [19]. This variation can be due to differences in patient population, CRRT practices, and blood transfusion threshold.

Most CRRT hemofilters are made to last for 72 hours. For many reasons, they frequently last for less time, which may interfere with treatment effectiveness and lead to increased cost. In a study that assessed patients with acute liver failure or decompensated chronic liver disease complicated by AKI requiring CRRT, the overall median life span was 9 hours (range, 6–16), in spite of apparently lower platelet counts on ICU admission [8]. Nevertheless, thrombocytopenia was an ominous predictor of both longer life span and bleeding complications [8]. Another study of 54 ICU patients on CRRT found an average filter life of 29.7 hours [20]. In our study, we found that the average and the median life span of CRRT filters were 23 hours and 20 hours, respectively. Surprisingly, there was no significant difference in hemoglobin level and PRBC transfusion between patients who had filter life span of <20 hours and ≥20 hours in up to 7 days of CRRT. We should note that the two patient groups had similar platelet count and coagulation profile.

Even though citrate is recommended as the first-line anticoagulant for CRRT [10], UFH is the most commonly used anticoagulant during CRRT to avoid clotting and to prolong filter life span. Its advantages include the wide experience with its use among physicians, short half-life, and the availability of an inhibitor [21]. Its drawbacks are its unpredictable pharmacokinetics, bleeding risk, and development of heparin-induced thrombocytopenia [21]. The effect of heparin infusion on CRRT filter life span is unclear. Some studies showed no correlation between the amount of heparin the patients received and the mean survival of the filters [20, 22]. A prospective observational study compared two groups of patients (circuit life span <48 hours versus >48 hours) and found that hematocrit, albumin levels, platelet count, INR, aPTT, and the dose of heparin were not significantly associated with circuit life [20]. Another study compared 40 filters of 12 ICU patients not anticoagulated because of high bleeding risk with 40 filters in 14 patients treated with low-dose prefilter heparin infusion and found that the mean circuit life was longer in the first group (32 hours; 95% CI, 20–44.4) than the second group (19.5 hours; 95% CI, 14.2–23.8; *p*=0.02) [23]. The mean heparin dose received heparin was 716 IU (95% CI, 647–785) [23]. Circuit lifespan did not correlate with INR, aPTT, or platelet count [23]. In our study, the use of IV heparin was not associated with longer filter life span. We should note that, during the study period, UFH was not administered through a structured protocol. When heparin is used for anticoagulation during CRRT, the filter is primed with 5000 IU, followed by a maximum loading dose of 5000 IU and a maintenance dose of 5–10 IU/kg/h [24]; although there is variation in practice [25], the use of heparin using an algorithm is thought to be more effective than fixed doses and has been associated with a filter life of approximately 20 hours in an audit in the United Kingdom [26]. Of note, our hospital did not use regional citrate for anticoagulation. Regional citrate may be more effective than UFH [24, 27, 28]; however, it is more difficult to use. The use of enoxaparin for anticoagulation during CRRT has been associated with longer filter lifespan compared with UFH (*p*=0.02) [7]. However, enoxaparin in AKI is rarely used because of the increased risk of bleeding.

The Kidney Disease Improving Global Outcomes (KDIGO) guidelines for AKI recommend the use of right jugular vein as the first choice and femoral vein as a second choice as a hemodialysis access [10]. Multiple studies showed significantly better filter life with femoral vein access in comparison to the internal jugular vein [29, 30]. A systematic review found that femoral access was associated 27% (95% CI, −4% to 69%; *p*=0.09) increase in filter survival compared with IJ access [31]. However, we found the hemodialysis venous access, femoral compared with internal jugular, was not associated with a longer filter life span (*p*=0.98).

In the current study, AKI patients requiring CRRT had higher ICU and hospital mortality rates compared with ICU patients not requiring this therapy, which has been demonstrated in several studies [32]. The mortality was similar in patients with shorter versus longer filter life span.

The results of this study should be interpreted in the light of its strength and limitations. It strengths include the relatively large sample size, including the large number of circuit filters, compared with other studies [6, 23]. The retrospective study design is being conducted in one tertiary-care hospital limit generalizability of results. Other limitations include the lack of data about the bleeding risk at CRRT start, the amount of blood loss when a filter clotted, and prefilter heparin effect on the anticoagulation profile.

In conclusion, our study found that anemia and transfusion requirement were common in critically ill patients requiring CRRT for AKI and that CRRT itself was an independent predictor of drop in hemoglobin to <70 g/L. The average CRRT filter life span was relatively short, but the hemoglobin level and blood transfusion requirement were not different in patients with shorter versus longer filter life span. Neither the use of prefilter heparin at small doses nor the location of access was associated with the life span of the filter. Anticoagulation using an UFH algorithm or regional citrate is recommended to prolong the filter life span.

## Figures and Tables

**Table 1:** Characteristics of all patients enrolled in the study, where cases were patients who required continuous renal replacement therapy in the intensive care unit and controls were critically ill patients who did receive renal replacement therapy. Cases and controls were matched for age, gender, APACHE II score, and admission category.

Variables	All patients (*N* = 197)	CRRT (*N* = 95)	No CRRT (*N* = 102)	*p* value
Age (years), mean ± SD	61.4 ± 16.4	55.7 ± 15.3	63.0 ± 17.3	0.15
Male gender, *N* (%)	121 (61.4)	60 (62.5)	61 (60.4)	0.76
Body mass index (kg/m^2^), mean ± SD	31.5 ± 19.4	33.6 ± 26.6	29.3 ± 7.7	0.12
APACHE II score, mean ± SD	25.6 ± 6.1	25.9 ± 7.1	25.4 ± 7.5	0.63
Admission type, *N* (%)				0.14
Medical	176 (89.3)	90 (93.8)	86 (85.1)	
Surgical	15 (7.6)	4 (4.2)	11 (10.9)	
Trauma	6 (3.0)	2 (2.1)	4 (4.0)	
Chronic illnesses, *N* (%)				
Cardiac comorbidity	83 (42.1)	47 (49.0)	36 (35.6)	0.06
Respiratory comorbidity	43 (21.8)	21 (21.9)	22 (21.8)	0.99
Hepatic comorbidity	12 (6.1)	9 (9.4)	3 (3.0)	0.06
Renal comorbidity	42 (21.3)	28 (29.2)	14 (13.9)	0.009
Immunocompromised state	18 (9.1)	8 (8.3)	10 (9.9)	0.70
Diabetes	116 (58.9)	61 (63.5)	55 (54.5)	0.20
Hypertension	122 (61.9)	62 (64.6)	60 (59.4)	0.45
Sepsis on admission, *N* (%)	50 (25.4)	34 (35.4)	16 (15.8)	0.002
Shock on admission, *N* (%)	37 (18.8)	27 (28.1)	10 (9.9)	0.001
Requirement for mechanical ventilation, *N* (%)	181 (91.9)	90 (93.8)	91 (90.1)	0.35
Laboratory findings				
Serum creatinine on admission (micromol/L), mean ± SD	175.7 ± 172.1	237.6 ± 213.8	116.8 ± 86.5	<0.001
Albumin on admission (g/L), mean ± SD	28.9 ± 5.9	29.0 ± 5.3	28.9 ± 6.4	0.98
Lactate on admission (mmol/L), mean ± SD	2.7 ± 2.8	3.3 ± 3.4	2.1 ± 1.8	0.002
Platelet count on admission (×10^9^/L), mean ± SD	233.7 ± 141.5	199.2 ± 117.4	266.5 ± 154.7	0.001
Hemoglobin on admission (g/L), mean ± SD	112.7 ± 23.1	111.8 ± 18.7	113.6 ± 26.6	0.60

APACHE: Acute Physiology and Chronic Health Evaluation; SD: standard deviation.

**Table 2:** Characteristics of patients on continuous renal replacement therapy for acute kidney injury categorized according to the median of the filter life span (20 hours).

	Filter life span <20 hours (*N* = 44)	Filter life span ≥20 hours (*N* = 51)	*p* value
Age (years), mean ± SD	58.1 ± 16.3	60.5 ± 14.1	0.45
Male gender, *N* (%)	25 (56.8)	34 (66.7)	0.32
Body mass index (kg/m^2^), mean ± SD	38.1 ± 38.4	29.9 ± 7.5	0.14
APACHE II score, mean ± SD	26.2 ± 7.1	25.8 ± 7.1	0.79
Admission type, *N* (%)			0.17
Medical	43 (97.7)	46 (90.2)	
Surgical	0 (0)	4 (7.8)	
Trauma	1 (2.3)	1 (2.0)	
Chronic illnesses, *N* (%)			
Cardiac comorbidity	24 (54.5)	22 (43.1)	0.27
Respiratory comorbidity	9 (20.5)	11 (21.6)	0.89
Hepatic comorbidity	3 (6.8)	6 (11.8)	0.50
Renal comorbidity	12 (27.3)	16 (31.4)	0.66
Immunocompromised state	4 (9.1)	4 (7.8)	0.56
Diabetes	29 (65.9)	31 (60.8)	0.61
Hypertension	28 (63.6)	33 (64.)	0.91
Sepsis on admission, *N* (%)	12 (27.3)	22 (43.1)	0.11
Shock on admission, *N* (%)	10 (22.7)	17 (33.3)	0.25
Requirement for mechanical ventilation, *N* (%)	40 (90.9)	49 (96.1)	0.30
Prefilter heparin use, *N* (%)	17 (38.6%)	15 (29.4%)	0.34
Location of hemodialysis access			0.98
Femoral vein	26 (59.1%)	31 (60.8%)	
Internal jugular vein	17 (38.6%)	19 (37.3%)	
Other	1 (2.3)	1 (2.0)	
Laboratory findings			
Serum creatinine on admission (micromol/L), mean ± SD	263.2 ± 228.3	286.1 ± 243.6	0.32
Serum creatinine on CRRT start (micromol/L), mean ± SD	366.0 ± 228.3	286.1 ± 169.0	0.06
Serum creatinine highest (micromol/L), mean ± SD	469.0 ± 243.6	367.6 ± 183.6	0.23
Albumin on admission (g/L), mean ± SD	29.4 ± 5.4	28.5 ± 5.3	0.42
Lactate on admission (mmol/L), mean ± SD	3.2 ± 4.1	3.4 ± 2.8	0.78
Platelet count on admission (×10^9^/L), mean ± SD	206.9 ± 105.5	192.5 ± 128.6	0.56
Hemoglobin on admission (g/L), mean ± SD	113. 9 ± 19.1	110.31 ± 18.5	0.36
Highest INR during CRRT, mean ± SD	2.2 ± 1.6	2.3 ± 1.5	0.73
Highest aPTT during CRRT (seconds), mean ± SD	83.2 ± 56.6	84.6 ± 46.6	0.89
Lowest platelet during CRRT (×10^9^/L), mean ± SD	95.8 ± 84.5	83.4 ± 71.6	0.45
Number of PRBC units transfused during CRRT, mean ± SD	2.5 ± 4.3	2.7 ± 3.8	0.81
Number of platelet units transfused during CRRT, mean ± SD	6.0 ± 13.3	63 ± 12.7	0.90
Number of FFP units transfused during CRRT, mean ± SD	3.1 ± 6.3	5.4 ± 11.3	0.24

APACHE: Acute Physiology and Chronic Health Evaluation; aPTT: activated partial thromboplastin time; CRRT: continuous renal replacement therapy; FFP: fresh frozen plasma; INR: international normalized ratio; PRBC: packed red blood cell; SD: standard deviation.

**Table 3:** Outcomes of patients on continuous renal replacement therapy for acute kidney injury categorized according to the median of the filter life span (20 hours).

	Filter life span <20 hours (*N* = 44)	Filter life span ≥20 hours (*N* = 51)	*p* value
Bleeding complication	5 (11.4)	5 (10.0)	0.83
ICU mortality, *N* (%)	22 (50)	30 (58.8)	0.39
Hospital mortality, *N* (%)	25 (56.8)	33 (64.7)	0.43
Mechanical ventilation duration (days), mean ± SD	23.8 ± 31.2	19.5 ± 19.1	0.44
ICU length of stay (days), mean ± SD	27.9 ± 32.4	25.0 ± 17.6	0.61
Hospital length of stay (days), mean ± SD	58.9 ± 86.3	61.5 ± 66.6	0.87

ICU: intensive care unit; SD: standard deviation.

## Data Availability

The data used to support the findings of this study are available from the corresponding author upon request.
